# Quercetin Downregulates Cyclooxygenase-2 Expression and HIF-1*α*/VEGF Signaling-Related Angiogenesis in a Mouse Model of Abdominal Aortic Aneurysm

**DOI:** 10.1155/2020/9485398

**Published:** 2020-08-22

**Authors:** Lian Wang, Haiwei Wu, Lei Xiong, Xiaolong Liu, Nan Yang, Liguo Luo, Tao Qin, Xian Zhu, Zhonghua Shen, Hua Jing, Jinming Chen

**Affiliations:** ^1^Department of Thoracic Surgery, The Second Affiliated Hospital, Zhejiang University School of Medicine, Hangzhou 310009, China; ^2^Department of Cardiothoracic Surgery, Jingling Hospital, Clinical Medicine School of Nanjing University, Nanjing 210002, China; ^3^Department of Cardiac Surgery, The Second Affiliated Hospital, Zhejiang University School of Medicine, Hangzhou 310009, China; ^4^Department of Anorectal Surgery, The Third People's Hospital of Hangzhou, Hangzhou 310009, China

## Abstract

**Objective:**

Abdominal aortic aneurysm (AAA) development has been characterized by increased expression of vascular endothelial growth factor (VEGF), which contributes to angiogenesis via cyclooxygenase-2 (COX-2). Quercetin, one of the most common and well-researched flavonoids and abundant in vegetables and fruits, has beneficial effects in inhibiting angiogenesis. This study investigated the antiangiogenic effects of quercetin on experimental aneurysms.

**Methods:**

We utilized the in vivo AAA mouse model induced by the periaortic application of CaCl_2_ to examine the effectiveness of quercetin in blocking angiogenesis. Quercetin was administered at 60 mg/kg once daily on the day of the AAA induction and then continued for 6 weeks. Celecoxib, a selective COX-2 inhibitor, was used as the positive control.

**Results:**

Our results demonstrated that quercetin significantly attenuated aneurysm growth in AAA mice and medial neovascularization. Accordingly, quercetin decreased the expression of proangiogenic mediators, including VEGF-A, intercellular adhesion molecule-1, vascular cell adhesion molecule 1, and vascular endothelial cadherin. Quercetin treatment also inhibited the expression of COX-2 and hypoxia-inducible factor 1*α* (HIF-1*α*). It was also found that quercetin-3-glucuronide, a major quercetin metabolite, downregulated the expression of COX-2, HIF-1*α*, VEGF-A, and matrix metalloproteinase activities in aortic vascular smooth muscle cells isolated from AAA mice.

**Conclusion:**

Quercetin attenuates neovascularization during AAA growth, and this effect is mediated via the inhibition of COX-2, which decreases HIF-1*α*/VEGF signaling-related angiogenesis.

## 1. Introduction

Abdominal aortic aneurysm (AAA) is an important cause of morbidity among males older than 65 years. Most aneurysms detected by screening are of small size and do not need immediate surgical intervention [1]. Currently, there is no effective treatment option. However, the overall mortality rate of ruptured AAA is almost 90% [1]. Therefore, the development of a viable medication to retard the growth of small aortic aneurysms has received considerable interest.

The local persistent inflammation, oxidative stress, and proteolysis in the aortic wall are main pathophysiological mechanisms of AAA progression [2]. An early report demonstrated that AAA is associated with marked angiogenic response at the periaortic region, which is related to the inflammatory infiltrate [3]. Angiogenesis, oxidative stress, and inflammation are key processes acting simultaneously and synergistically for the maintenance of AAA [2]. Moreover, these neovessels increased extracellular matrix (ECM) degradation through matrix metalloproteinases (MMPs) in the AAA wall, which could substantially contribute to aneurysm wall degeneration and rupture [4]. It is well documented that the suppression of angiogenesis can be a substantial strategy for the AAA treatment [5–8]. Human AAA specimen contains high levels of vascular endothelial growth factor (VEGF) and VEGF receptors [9]. VEGF belongs to the platelet-derived growth factor supergene family, regulates endothelial regeneration, and plays essential roles in pathological angiogenesis [10]. Furthermore, recombinant human VEGF enhances the angiotensin II-induced AAA formation in apolipoprotein E-deficient mice [11], suggesting that increased expressions of VEGF and their receptors may impact different pathways involved in the AAA etiology. In a mouse model of CaCl_2_-induced AAA, inhibiting VEGF-A signaling suppressed angiogenesis, in association with reduced MMP activities, ECM degradation, and aneurysm growth [5]. Therefore, inhibiting VEGF-driven angiogenesis may offer a valid medical therapy against aortic aneurysm progression.

It was reported that various natural products including resveratrol [6], curcumin [12], ginkgo biloba extract [13], polyunsaturated fatty acids [14], and taurine [15] could partially inhibit the development of AAA. We have reported in previous papers that quercetin, an important natural product and a ubiquitous flavonoid with anti-inflammatory activity, suppresses the development of AAA via inhibiting the inflammatory response and the oxidative stress in the mouse AAA model [16, 17]. Many previous studies have also documented the inhibitory effects of quercetin on cyclooxygenase-2 (COX-2) expression [18] and angiogenesis [19]. Moreover, the AAA development has been characterized by the increased expression of COX-2, and the inactivation of COX-2 may lead to a new approach to prevent AAA expansion [20–22]. It is worth mentioning that rigorous analyses reveal that COX-2 upregulation is transcriptional and is associated with the hypoxia-inducible factor 1*α* (HIF-1*α*) induction [23]. Furthermore, HIF-1*α* activates VEGF expression by directly binding to VEGF promoter during hypoxia in mammalian cells [24]. In atherosclerosis, an independent risk marker for AAA [25], localized hypoxia and oxidative stress in the vessel wall trigger the activation of HIF-1*α* and the expression of VEGF [26]. Therefore, the purpose of this study was to determine whether the antiangiogenic properties of quercetin are associated with the attenuation of experimental aneurysm formation in the AAA model.

## 2. Materials and Methods

### 2.1. Ethics Statement

This study was carried out in accordance with the Guide for the Care and Use of Laboratory Animals (National Institutes of Health, Washington DC, 1996). The protocol was approved by the Animal Care and Use Committee (Nanjing University, Nanjing, China). All efforts were made to minimize the number of animals used and to ensure minimal suffering.

### 2.2. Experimental AAA Induction

Male C57/BL6 mice (5-6 weeks of age) were purchased from Vital River Laboratory Animal Technology (Beijing, China). AAA was induced in the infrarenal abdominal aorta by the periaortic application of CaCl_2_ as described previously [16]. During the operation, the external diameter of the aorta was measured with a digital camera (HDR-SR12E, Sony, Tokyo, Japan) at approximately the center of the section between the left renal artery and the iliac bifurcation. NaCl (0.9%) was substituted for CaCl_2_ in sham operation animals. Six weeks later, heart rate and mean arterial pressure were monitored by the computerized tail-cuff system with four-channel mouse platform (BP-2000, Visitech Systems, Inc., Apex, NC), according to the manufacturer's instructions. The mice were laparotomized, and the aortic diameters were measured again. The presence of an AAA is defined as a dilatation of the aorta more than 1.5 times its original diameter [1]. The animals were then sacrificed by the left heart injection of potassium chloride, and the aortic tissues were collected.

### 2.3. Drug Administration

Four groups of mice (*n* = 20/group) were created: sham operation (sham group), AAA plus vehicle treatment (control group), AAA plus quercetin treatment (AQ group), and AAA plus celecoxib treatment (AC group). Quercetin (Sigma-Aldrich, Shanghai, China) and celecoxib (Celebrex®, Pfizer, NY) were suspended into 0.5% sodium carboxymethyl cellulose (SCMC, Sinopharm Chemical Reagent, Shanghai, China), and the fresh solutions were prepared every other day. Quercetin (60 mg/kg), celecoxib suspension (50 mg/kg), or an equal volume of SCMC was administered orally to mice by gavage. Drug treatments were started postoperatively on the day of operation and continued for 6 weeks.

### 2.4. Histology and Immunostaining

The infrarenal abdominal aorta segments (*n* = 5 in each group) were fixed with 10% formalin solution and imbedded in paraffin. Aortic tissue sections were stained with Victoria blue (VB) for elastin and Masson's trichrome (MT) for collagen. VB-stained sections were evaluated for qualitative changes and elastin loss. Immunohistochemical staining with CD31 antibody was used as an early indicator of the endothelial cell differentiation during angiogenesis. As described previously [16], the slides were incubated with a rabbit antibody against CD31 (1 : 200, A01513-1, Boster, Wuhan, China) and subsequently horseradish peroxidase-conjugated goat antibodies against rabbit (sc-3837, Santa Cruz Biotechnology, Santa Cruz, CA, US). The slides were examined and photographed using an Olympus BX–51 microscope equipped with a digital camera. Images were analyzed by the ImageJ (FIJI-64 bit) software (National Institutes of Health, MD, US). CD31-positive vessels were quantified by counting the vessels in 20 grid fields and graphed. The slides were evaluated by 3 independent observers blinded to the groups.

### 2.5. Prostaglandin E2 (PGE_2_) Enzyme-Linked Immunosorbent Assay (ELISA)

COX-2 has been found to be widely expressed in the aneurysmal wall with concomitant synthesis of PGE_2_, which is a commonly used method for the detection of COX-2 modulation during the AAA development [27]. The concentrations of PGE_2_ in aortic tissues were determined using a commercial competitive ELISA kit (514010, Cayman, Ann Arbor, MI) according to the manufacturer's instructions. Results were expressed as pg/ml.

### 2.6. *In Vitro* Studies Using Vascular Smooth Muscle Cells (VSMCs) Isolated from AAA Mice

Aortas isolated from AAA mice (*n* = 10) were made free of the surrounding fatty tissues and cut longitudinally. A scalpel was used to remove the endothelial cell layers and the adventitia, and the remaining medial layer was washed with phosphate-buffered saline (PBS) and chopped into 5 × 5 mm pieces. The tissue slices were enzymatically degraded (30 min at 37°C) in a mixture of 125 U/mg collagenase and 3 U/mg elastase, which was prepared in serum-free Dulbecco's modified Eagle's medium (DMEM) F12 culture medium (Invitrogen, Carlsbad, CA). The digestates were then centrifuged at 400 g for 5 min to remove the enzymes. The pelleted tissue pieces were then cultured in T-75 flasks with DMEMF12 containing 10% fetal bovine serum for over 15 days. VSMCs between passages 2 and 5 were then seeded onto six-well tissue culture plates at a density of 3 × 10^4^ cells/well and cultured in DMEMF12 culture medium supplemented with 10% fetal calf serum, Hepes 20 mmol/l, and antibiotics. Cultures were maintained at 37°C in a humid atmosphere of 5% CO_2_. Primary aneurismal VSMCs derived by outgrowth from these tissue explants were cultured for over 2 weeks, and the cells passaged when confluence was attained. Cells were starved overnight and then treated with 50 *μ*M quercetin-3-O-glucuronide (Q3GA, Sigma-Aldrich, Shanghai, China), the major circulating quercetin metabolite, or 20 *μ*M celecoxib for 72 hours and then harvested for total RNA isolation and analysis.

### 2.7. Quantitative Real-Time Reverse Transcription Polymerase Chain Reaction (qRT-PCR)

qRT-PCR was used to define the messenger RNA (mRNA) expression of VEGF-A, intercellular adhesion molecule-1 (ICAM-1), vascular cell adhesion molecule 1 (VCAM-1), vascular endothelial cadherin (VE-cadherin), transforming growth factor-1 (TGF1), fibroblast growth factor (FGF), COX-2, HIF-1*α*, MMP-2, and MMP-9. Total RNA was extracted using a TRIzol reagent (Invitrogen), and 1 *μ*g total RNA was reverse transcribed into cDNA using the AMV First-Strand cDNA Synthesis Kit (Invitrogen, USA). Gene transcripts were quantified by real-time RT-PCR using the ABI SYBR Green PCR Master Mix (Thermo Fisher) and the ABI StepOnePlus system according to the manufacturer's protocol. Data was analyzed with the StepOnePlus software. The constitutive expression gene, GAPDH, was used as an internal control to verify the fluorescent RT-PCR reaction. Primers were synthesized by Sangon Biological Engineering Technology and Services (Shanghai, China). The primer sequences are listed in Table 1.

### 2.8. Immunoblotting

Protein expressions were determined by Western blot analysis as described in detail previously [16]. Primary antibodies for VEGF-A (512902, BioLegend, CA, USA) and HIF-1*α* (ab82832, Abcam, MA, USA) were added and incubated. All blots were incubated with anti-*β*-actin antibody (4970S, Cell Signaling Technology, MA, US) to confirm protein loading levels. Quantification of immunoblots was carried out using ImageJ.

### 2.9. *In Situ* Gelatin Zymography

The MMP activities of the cultured VSMCs were measured with zymography and quantified as we described previously [17].

### 2.10. Data Presentation and Statistical Analysis

The percent increase of the aortic diameter was calculated as [(6‐week aortic diameter − operation aortic diameter)/operation aortic diameter] × 100. Between-group comparisons were assessed using one-way analysis of variance (ANOVA) with Bonferroni correction. A paired Student's *t* test was used to compare the aortic diameter at the time of operation and sacrifice. Data are reported as mean ± standard deviation. AAA incidence between groups was analyzed using Fisher's exact test. SPSS for Windows version 17.0 (SPSS Inc., Chicago, IL, US) was used for statistical calculations. Significance was assumed at *P* value < 0.05.

## 3. Results

### 3.1. General Condition and Aneurysm Size

**Figure 1:**
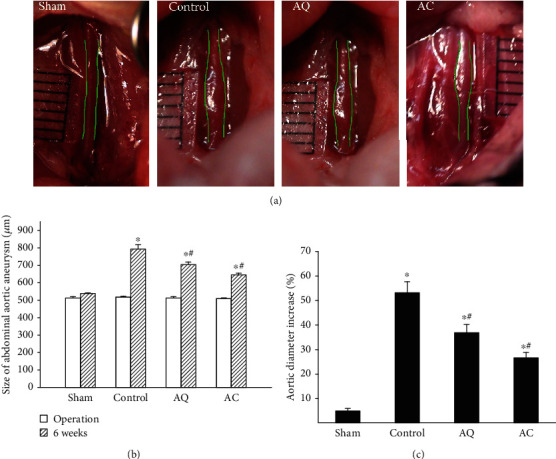
The effect of quercetin and celecoxib on the development of CaCl_2_-induced AAAs in mice. (a) Representative photographs showing macroscopic features of the aorta treated with saline or CaCl_2_ followed by quercetin (AQ) or celecoxib (AC). Each division in the ruler scale represents 500 *μ*m. (b) The aortic diameters were measured before the AAA induction and on postoperative 6 weeks. Quercetin and celecoxib significantly inhibited aneurysm expansion as compared with controls. (c) The percentage increase in aortic diameter was significantly less in quercetin- and celecoxib-treated mice (*n* = 20 per group). ^∗^*P* < 0.05 vs. sham, ^#^*P* < 0.05 vs. control.

No changes in general behavior of mice from all groups were observed. All mice survived to postoperative 6 weeks, when the animals were euthanized. There was no difference in body weight, heart rate, or mean arterial pressure among the four groups (Supplementary Table 1). Baseline aortic sizes were similar among the groups at operation. However, at relaparotomy on 6 weeks later, aortic diameters were statistically different among the groups (Figures 1(a) and 1(b)). Compared to control mice, quercetin-treated mice had a smaller mean aortic size. Similar inhibitory profiles were seen in celecoxib-treated mice. The percentage increase in aortic diameter was significantly less in quercetin- and celecoxib-treated groups, compared with the aneurismal expansion seen in controls (Figure 1(c)). Both the quercetin and celecoxib treatment significantly decreased the AAA incidence versus the control group (Supplementary Table 1).

### 3.2. Structure of the Aortic Wall

**Figure 2:**
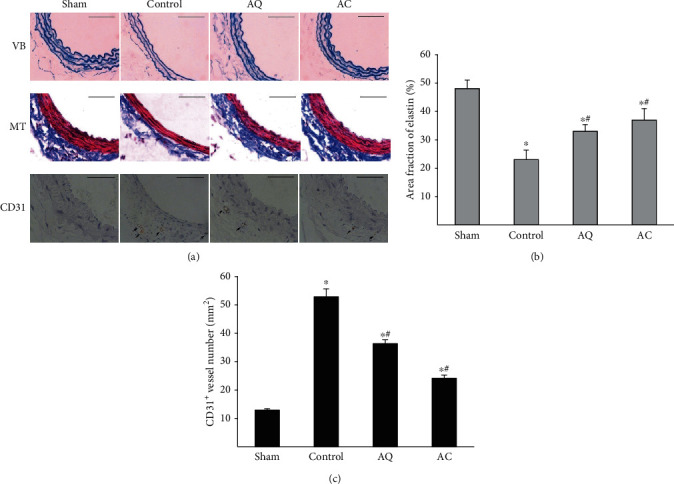
The effect of quercetin and celecoxib on histology and CD31^+^ cells of CaCl_2_-induced AAAs. (a) Transverse sections of formalin-fixed aortic tissue were stained with Victoria blue (VB) (elastin in blue) and Masson's trichrome (MT) stain (collagen in blue). Sections were immunostained for CD31 to examine the microvessel density. Arrows indicate positive CD31 immunostaining. Scale bars are 50 *μ*m. Each section shown is a representative of five samples with similar results. (b) Percentage of area positive for elastic fibers. The area of elastic fibers in VB-stained sections was calculated by quantitative morphometric analysis with ImageJ. Results were expressed as a percentage of the area of the aortic media. (c) CD31^+^ microvessel contents in the mouse aortic wall (*n* = 5 in each group). ^∗^*P* < 0.05 vs. sham, ^#^*P* < 0.05 vs. control.

VB staining demonstrated the flattening and destruction (fracturing and loss) of the elastic fibers in control mice compared with the sham operation (Figure 2(a)). Scoring of elastin preservation showed a significant decrease in controls. Treatment with quercetin and celecoxib significantly inhibited the proteolysis of elastin in AAA (Figures 2(a) and 2(b)). MT staining showed that the collagen (another major constituent of ECM components) content of the aortic wall in control animals was smaller than that in the sham group. Treatment with quercetin and celecoxib limited the loss of collagen in the aortic wall (Figure 2(a)).

### 3.3. Medial Neovascularization and Expression of Proangiogenic Mediator

**Figure 3:**
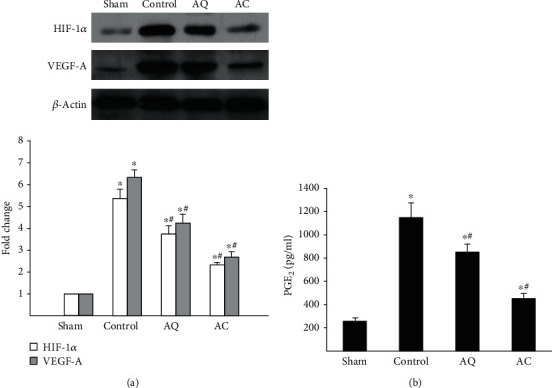
The expression of HIF-1*α* and VEGF-A and the quantification of PGE_2_. (a) Western blot for HIF-1*α* and VEGF-A expression. Beta-actin signal served as an internal control of protein loading. Densitometric analyses are shown beneath the representative blots. Data were expressed as fold changes compared with values in sham animals. (b) The effect of quercetin and celecoxib on the PGE_2_ synthesis. PGE_2_ levels were measured by ELISA (*n* = 5 per group). ^∗^*P* < 0.05 vs. sham, ^#^*P* < 0.05 vs. control.

Immunohistochemical staining showed increased CD31-positive microvessel densities in the medial layer of the aorta from control animals. The numbers of CD31-positive vessels were decreased significantly in the AQ and AC groups, compared with controls (Figures 2(a) and 2(c)). Moreover, the mRNA expression of proangiogenic cytokines including VEGF-A, ICAM-1, VCAM-1, and VE-cadherin was significantly upregulated in control mice (Table 2). Quercetin and celecoxib decreased the expression of VEGF-A, ICAM-1, VCAM-1, and VE-cadherin. Furthermore, quercetin- and celecoxib-treated mice also showed the reduced protein expression of VEGF-A (Figure 3(a)). There was no significant difference in gene expressions of TGF1 or FGF among the groups (Table 2).

### 3.4. COX-2 and HIF-1*α* Expression

Table 2 shows that the mRNA expression of COX-2 and HIF-1*α* was significantly upregulated in control mice. Quercetin and celecoxib decreased the gene expression of COX-2 and HIF-1*α*. These effects were confirmed by the simultaneous analysis of protein levels using immunoblotting and ELISA, respectively (Figures 3(a) and 3(b)).

### 3.5. *In Vitro* Studies

**Figure 4:**
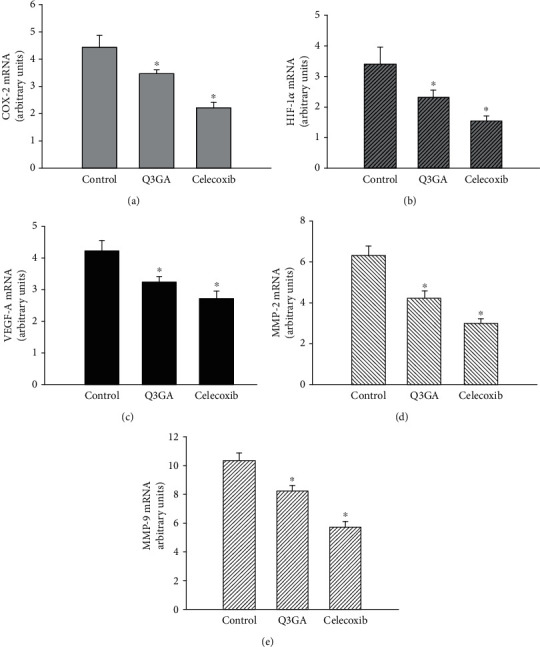
In vitro gene expression of (a) COX-2, (b) HIF-1*α*, (c) VEGF-A, (d) MMP-2, and (e) MMP-9 in cultured VSMCs. All signals were normalized with GAPDH and presented in arbitrary units (*n* = 5 per group). ^∗^*P* < 0.05 vs. the control group.

**Figure 5:**
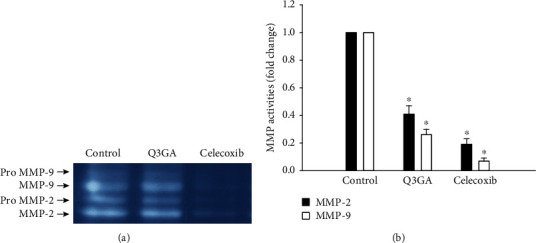
Gelatin zymographic analysis of MMP activities after in vitro incubation with VSMCs. (a) Representative images of gelatin zymography showing the decrease of MMP activities in the Q3GA and celecoxib groups. (b) Bar graphs show the quantification of MMP-2 and MMP-9 activities, respectively (*n* = 5). ^∗^*P* < 0.05 vs. the control group.

Finally, to examine the role of quercetin on angiogenesis in vitro, we used aortic VSMCs isolated from AAA mice. As shown in Figure 4, COX-2, HIF-1*α*, and VEGF-A were downregulated under the Q3GA and celecoxib treatment. Then, we assayed MMP expression, which is an essential step in the angiogenesis process. Consistent with the negative regulation of VEGF-A gene expression, we also observed a significant downregulation of MMP-2 and MMP-9, in response to the Q3GA or celecoxib treatment (Figure 4). MMP activities were measured in VSMCs derived from AAA. In line with qRT-PCR results, gelatin zymography showed that Q3GA and celecoxib decreased MMP-2 and MMP-9 activities (Figure 5).

## 4. Discussion

Angiogenesis, the formation of new blood vessels from preexisting vessels, plays an important role in many physiological and pathological processes. As we observed in the present study, adventitial microvessel density was increased during the CaCl_2_-induced AAA formation. Indeed, substantial evidence implicates the pathological neovascularization as one of the key components of the pathophysiology of the AAA formation and progression [4]. These microvessels are strongly associated with increased vessel permeability, consequent accumulation of inflammatory cells, and increased MMP expression, which would contribute to proteolytic degradation of ECM. The result is progressive destruction of the aortic wall and ultimately aneurysm expansion and possible rupture. Accordingly, there has been a growing interest in neovascularization as a possible therapy target for the prevention or treatment of aneurysm. In this regard, emerging evidence indicated that antiangiogenic therapy could suppress the formation and progression of experimental AAA [5–8]. Interestingly, there are several lines of evidence demonstrating that quercetin, the most abundant form of the flavonoids, can have antiangiogenic effects in a range of human tumor cell lines [28]. More importantly, quercetin is a major flavonoid that can be found in a wide range of fruits and vegetables, and daily intake of quercetin in foods is ethnopharmacologically meaningful and beneficial to cardiovascular disease [29]. However, an association between quercetin and angiogenesis has not been studied in aneurysm. Our findings here reveal a novel action of quercetin and suggest a potential use for this compound in the treatment of angiogenesis-mediated diseases such as AAA.

Several studies have demonstrated that the vascular protective effects of quercetin are associated with anti-inflammatory benefits and potentially also influence oxidative stress. Our previous reports have shown that aneurysmal degeneration can be suppressed by quercetin and that the mechanisms underlying the protective effects of quercetin consist of its anti-inflammatory and antioxidative properties [16, 17]. In the present study, we extended these observations to demonstrate for the first time that quercetin can inhibit angiogenesis during the AAA formation, accompanied by the reduction of COX-2, HIF-1*α*, and VEGF expression. Our data provide experimental evidence that quercetin has antiangiogenic effects both in vitro and in vivo, and this could be one of the mechanisms by which quercetin restricts aneurysm growth.

A complex chronic inflammatory process including macrophages, lymphocytes, neutrophils, and VSMCs is another character of AAA [2]. The infiltrating inflammatory cells and VSMCs could secrete various mediators including PGE_2_. PGE_2_ increases the production of MMPs [30], which are suggested to play a prominent role in the degradation of the vascular wall as mentioned previously. Furthermore, the increased synthesis of PGE_2_ in aneurysmal tissue may then recruit additional inflammatory cells and promote innate and adaptive immune responses, leading to continued aneurysmal degeneration. Therefore, the selective inhibition of PGE_2_ synthesis could be an effective treatment to the aortic aneurysm expansion [27]. As our study showed, the PGE_2_ production was markedly decreased in the mice treated with quercetin, which was correlated with inhibited aneurysmal dilation and decreased MMP activities. The local PGE_2_ formation is synthesized by COX-1 and COX-2. COX-1 is generally known as a basal enzyme, whereas COX-2 is encoded by an immediate-early gene. COX-2 can be upregulated by various proinflammatory agents and may play an important role in the developing neovasculature under a hypoxic condition. It has been demonstrated that COX-2 is expressed widely in the aneurysm wall of human AAA [31], suggesting a primary role for COX-2 in the development of this disease. Moreover, COX-2 expression increased with the extent of inflammatory cell infiltration and the degree of human AAA neovascularization. Thus, the promotion of angiogenesis by COX-2 may play a role in the AAA development [32]. We describe here for the first time the strong expression of COX-2 in the CaCl_2_-induced AAA model and in medial VSMCs cultured from the aneurysm wall. It was demonstrated that the selective COX-2 inhibition with celecoxib attenuates the incidence and severity of angiotensin II-induced AAA formation in mice [33]. However, celecoxib did not reduce the expression of markers of macrophage-dependent inflammation in this model [21], but increased the expression of differentiated VSMC markers and reduced differentiation marker expression during AAA progression [20]. In this study, we compared the quercetin treatment versus the standard celecoxib treatment in the setting of experimental AAA. Quercetin significantly suppressed COX-2 expression both in vivo and in vitro. Although the effects of quercetin were less than those of celecoxib, they were not surprising. Quercetin attenuates the induction of COX-2 expression and activity and regulates its downstream pathways in different experimental models and cell cultures [34–36]. Furthermore, quercetin significantly suppresses COX-2 mRNA, protein expression, and PGE_2_ production, as well as COX-2-mediated angiogenesis in human endothelial cells in a dose-dependent manner [18]. Based on the data from these studies, our findings suggest that the COX-2 inhibition is one of the mechanisms explaining the inhibitory effect of quercetin on the aneurysm expansion.

There is an ample research demonstrating that COX-2 promotes angiogenesis via multiple mechanisms. For example, studies revealed that the increased expression of COX-2 closely correlates with enhanced VEGF mRNA or protein production. VEGF is one of the most potent angiogenic factors, which is capable of promoting endothelial cell proliferation and migration and inducing vascular permeability [10]. As we observed in our study, increased adventitial neovascularization was accompanied by the enhanced expression of COX-2, PGE_2_, and VEGF during the AAA formation. In fact, the activation of COX-2 in interstitial cells is necessary for the upregulation of VEGF, endothelial cell proliferation, and formation of new microvessels [37]. It was reported that extremely high levels of VEGF and VEGF receptors may play a significant role in the AAA etiology [5, 9]. On the other hand, exogenous recombinant human VEGF accelerates the expansion of angiotensin II-induced aneurysms. This effect is dependent on the increased gene expression of MMP-2 [11]. Furthermore, inhibiting VEGF signaling reduces MMP activities, ECM degradation, neoangiogenesis, and AAA development in mice [5]. Proteinases from the MMP system directly participate in the angiogenic process [38]. Therefore, we also evaluated the association of MMP expression with quercetin in CaCl_2_-induced AAA in the present study. We found that the quercetin blocks MMP signaling both in vitro and in vivo for the first time. These results partly explain the effect of quercetin on angiogenesis. Additionally, we have reported earlier that the quercetin treatment inhibits the reactive oxygen species generation in the aortic tissue from AAA mice [17]. Prior experiments have demonstrated that the augmented oxidative stress in arteries could be blunted by the COX-2 inhibition [39]. More importantly, oxidative stress may modulate neovascularization in several different ways but one of the most recognizable is increased VEGF expression [40]. When viewed together, our data strongly suggest that the antiangiogenesis effect of quercetin participates in its suppression of AAA via a synergistic combination of COX-2, VEGF, MMPs, and oxidative stress.

Another noteworthy finding of the present study is that enhanced angiogenesis was accompanied by the HIF-1*α*/VEGF signaling pathway upregulation in AAA mice. However, the quercetin treatment significantly decreased the pathway activation here in vivo and in vitro, as measured by the expression levels of HIF-1*α* and VEGF. The induction of VEGF is an essential component of blood vessel reactivity and angiogenesis under hypoxic conditions, and HIF is known to be a critical transcription factor of this process [10]. Accumulating studies have already demonstrated COX-2 upregulation during hypoxia together with HIF-1*α* and VEGF [41]. Exogenous PGE_2_, a principal component of COX-2 products, induces the expression of HIF-1*α*, a process which could be suppressed by a selective COX-2 inhibitor [42]. COX-2-expressing vector increases both proliferation and tube formation of human umbilical vein endothelial cells. These in vitro angiogenic effects can be reduced either by blocking VEGF or COX-2 inhibitor. Furthermore, HIF-1*α* increases concomitantly with VEGF after exogenous PGE_2_ stimulation, but this effect is blocked by the PGE_2_ receptor antagonist [41]. However, an association between COX-2 expression and HIF-1*α* with angiogenesis has not been studied in AAA. In this study, we evaluated the association between COX-2 expression and HIF-1*α* with angiogenesis in the AAA mouse model and VSMCs isolated from AAA. Our results reveal that quercetin exerts its suppressive effects on angiogenesis and this may be through the COX-2-mediated HIF-1*α*/VEGF pathway during the AAA formation. Nevertheless, we did not administer a HIF-1*α*- or VEGF-specific inhibitor because the complex events that occur under in vivo conditions are largely unknown. Knock-out mice can be a more powerful tool for determining the potential relationship in future study designs.

In our previous studies, the quercetin treatment was initiated 2 weeks before CaCl_2_-induced AAA and continued for 6 weeks. The incidence of AAA was lower compared to placebo [16, 17]. In the current experiment, we changed our quercetin administration regimen and found that the AAA formation was blocked even though it was given after the AAA induction. This means a timely pharmacological intervention using angiogenesis inhibitors (such as quercetin) may be beneficial in preexisting aneurysms. It is encouraging because there is no effective treatment option for small AAA in humans despite their gradual expansion. However, it is unknown whether these experimental findings can be extended to human AAA. Moreover, a single antiangiogenic drug may not be enough to combat the complex network of different angiogenic factors and the pathways involved in the angiogenic process in human AAA. It was anticipated that combinations with other drugs would be more effective rather than the quercetin monotherapy. In addition, this study did not investigate the effect of quercetin on lipid metabolism, which is an important risk factor for AAA. These limitations need to be addressed in future investigations.

## 5. Conclusions

The current study results demonstrated that the antiangiogenic effect and inhibitory mechanism of quercetin attenuate AAA in the CaCl_2_-induced mice. The antiangiogenic effect of quercetin is associated with decreased expression of COX-2. In addition, quercetin-induced aneurysm inhibition is accompanied by the reduced HIF-1*α*/VEGF signaling pathway. These results lend further support to existing data supporting quercetin as a possible therapeutic candidate in early AAA suppression.

## Figures and Tables

**Table 1:** The primer sequences for the qPCRs in Materials and Methods.

Gene	Forward	Reverse
VEGF-A	5′-GTAACGATGAAGCCCTGGAGT-3′	5′-TGTTCTGTCTTTCTTTGGTCTGC-3′
ICAM-1	5′-CTCGTGATGGCAGCCTCTTAT-3′	5′-GGCTTGTCCCTTGAGTTTTATG-3′
VCAM-1	5′-AGTGTTGCTTGGACTGACTGTTG-3′	5′-GACCTCTTTACCGTTTGCCTAT-3′
VE-cadherin	5′-ATCTCAGACAACGGCAATCC-3′	5′-GAAAATTGCCACCAGTGCTT-3′
TGF1	5′-GCCCAGCACTTTTTGATTACTA-3′	5′-AGGTTGATTGGTGTCTGAGCA-3′
FGF	5′-TTGTGTCTATCAAGGGAGTGTGTG-3′	5′-AGTATTTCCGTGACCGGTAAGTAT-3′
COX-2	5′-TGCTGGAAAAGGTTCTTCTACG-3′	5′-GAACCCAGGTCCTCGCTTAT-3′
HIF-1*α*	5′-AAAGAACTAAACACACAGCGGA-3′	5′-ACAAATCAGCACCAAGCACG-3′
MMP-2	5′-AAGGATGGACTCCTGGCACATGCCTTT-3′	5′-ACCTGTGGGCTTGTCACGTGGTGT-3′
MMP-9	5′-AAGGACGGCCTTCTGGCACACGCCTTT-3′	5′-GTGGTATAGTGGGACACATAGTGG-3′
GAPDH	5′-GGTTGTCTCCTGCGACTTCA-3′	5′-TGGTCCAGGGTTTCTTACTCC-3′

Abbreviations: VEGF-A: vascular endothelial growth factor-A; ICAM-1: intercellular adhesion molecule-1; VCAM-1: vascular cell adhesion molecule 1; VE-cadherin: vascular endothelial cadherin; TGF1: transforming growth factor-1; FGF: fibroblast growth factor; COX-2: cyclooxygenase-2; HIF-1*α*: hypoxia-inducible factor 1*α*; MMP: matrix metalloproteinase.

**Table 2:** Summary of gene expression profiles in aortic tissue. Results are presented in arbitrary units normalized to GAPDH rRNA and shown as mean ± standard deviation (*n* = 5 per group).

	Sham	Control	AQ	AC
VEGF-A	0.54 ± 0.04	2.94 ± 0.16^∗^	1.82 ± 0.20^∗^^#^	1.34 ± 0.10^∗^^#^
ICAM-1	1.04 ± 0.12	4.15 ± 0.09^∗^	2.87 ± 0.15^∗^^#^	3.14 ± 0.12^∗^^#^
VCAM-1	0.75 ± 0.04	3.47 ± 0.12^∗^	2.65 ± 0.16^∗^^#^	2.43 ± 0.19^∗^^#^
VE-cadherin	1.15 ± 0.10	5.42 ± 0.18^∗^	4.35 ± 0.09^∗^^#^	3.54 ± 0.20^∗^^#^
TGF1	1.46 ± 0.16	1.32 ± 0.09	1.41 ± 0.11	1.61 ± 0.22
FGF	0.53 ± 0.05	0.34 ± 0.15	0.41 ± 0.14	0.38 ± 0.12
COX-2	1.14 ± 0.13	3.67 ± 0.11^∗^	2.74 ± 0.15^∗^^#^	1.56 ± 0.09^∗^^#^
HIF-1*α*	0.95 ± 0.14	6.51 ± 0.39^∗^	4.85 ± 0.27^∗^^#^	3.23 ± 0.25^∗^^#^

Abbreviations: VEGF-A: vascular endothelial growth factor-A; ICAM-1: intercellular adhesion molecule-1; VCAM-1: vascular cell adhesion molecule 1; VE-cadherin: vascular endothelial cadherin; TGF1: transforming growth factor-1; FGF: fibroblast growth factor; COX-2: cyclooxygenase-2; HIF-1*α*: hypoxia-inducible factor 1*α*. ^∗^*P* < 0.05 vs. sham, ^#^*P* < 0.05 vs. control.

## Data Availability

All data of this study can be acquired from the author if necessary.

## Supplementary Materials

Supplementary Materials Table 1: body weight (BW) increase, heart rate (HR), mean arterial pressure (MAP), and abdominal aortic aneurysm (AAA) incidence after treatment with NaCl or CaCl_2_ followed by quercetin (AQ) or celecoxib (AC) (*n* = 20 per group).
