# Supplementary material for: Quercetin Downregulates Cyclooxygenase-2 Expression and HIF-1α/VEGF Signaling-Related Angiogenesis in a Mouse Model of Abdominal Aortic Aneurysm
Source: Biomed Res Int. 2020 Aug 22;2020:9485398. doi: 10.1155/2020/9485398 (PMC7463408; doi:10.1155/2020/9485398)
Supplement: Supplementary Materials — Table 1: body weight (BW) increase, heart rate (HR), mean arterial pressure (MAP), and abdominal aortic aneurysm (AAA) incidence after treatment with NaCl or CaCl2 followed by quercetin (AQ) or celecoxib (AC) (n = 20 per group). [file 9485398.f1.pdf]

SUPPLEMENTARY TABLE 1.

Body weight (BW) increase, heart rate (HR), mean arterial pressure (MAP) and abdominal aortic aneurysm (AAA) incidence after treatment with NaCl or $CaCl_2$ followed by quercetin (AQ) or celecoxib (AC) (n = 20 per group).

	Sham	Control	AQ	AC
BW increase (%)	15.3±2.1	14.8±2.2	15.1±1.5	14.9±1.9
HR (bpm)	633±25	630±28	627±23	635±30
MAP (mmHg)	85±8	86±9	85±7	83±6
AAA incidence (%)	0(0/20)	65%(13/20) *	25%(5/20) $^{*\#}$	15%(3/20) $^{*\#}$

BW and aortic diameter were measured before NaCl or $CaCl_2$ incubation (operation) and at sacrifice (6 weeks post-operation). The percent increase was represented as a percent compared with operation. AAA is defined as a dilatation of the aorta more than 1.5 times its original diameter. HR and MAP were measured by the tail-cuff system. Measurements were expressed as mean ± SD. * $P < 0.05$, compared with Sham, # $P < 0.05$, compared with Control mice.
